# Pathways to lung cancer diagnosis among individuals who did not receive lung cancer screening: a qualitative study

**DOI:** 10.1186/s12875-023-02158-7

**Published:** 2023-10-03

**Authors:** Rachel D. McCarty, Mollie E. Barnard, Katherine A. Lawson-Michod, Makelle Owens, Sarah E. Green, Samantha Derzon, Lea Karabegovic, Wallace L. Akerley, Melissa H. Watt, Jennifer A. Doherty, Laurie Grieshober

**Affiliations:** 1 Huntsman Cancer Institute, University of Utah, 2000 Cir of Hope Dr, Salt Lake City, UT 84112 USA; 2 Department of Population Health Sciences Spencer Fox Eccles School of Medicine, University of Utah Intermountain Healthcare, University of Utah, 295 Chipeta Way, Salt Lake City, UT 84108 USA; 3 Slone Epidemiology Center, Boston University Chobanian & Avedisian School of Medicine, 72 East Concord St, Boston, MA 02118 USA; 4 Department of Internal Medicine, Spencer Fox Eccles School of Medicine, University of Utah, 30 N 1900 E, Salt Lake City, UT 84132 USA; 5 San Antonio Military Medical Center Internal Medicine Residency, Brooke Army Medical Center, 3551 Roger Brooke Dr, San Antonio, TX 78234 USA; 6 Danbury Hospital Department of Surgery, Danbury Hospital, 24 Hospital Ave, Danbury, CT 06810 USA; 7 Intermountain Healthcare, Utah Valley Hospital, Utah Valley Family Medicine Residency, 475 W 940 N, Provo, Provo, UT 84604 USA; 8 Division of Oncology, Spencer Fox Eccles School of Medicine, University of Utah, 30 N 1900 E, Salt Lake City, UT 84132 USA

**Keywords:** Lung cancer, Pathways to diagnosis, Qualitative study, Models of pathways to treatment

## Abstract

**Background:**

Although early detection of lung cancer through screening is associated with better prognosis, most lung cancers are diagnosed among unscreened individuals. We therefore sought to characterize pathways to lung cancer diagnosis among unscreened individuals.

**Methods:**

Participants were individuals with lung cancer who did not undergo asymptomatic lung cancer screening (n = 13) and healthcare providers who may be involved in the pathway to lung cancer diagnosis (n = 13). We conducted semi-structured interviews to identify themes in lung cancer patients’ narratives of their cancer diagnoses and providers’ personal and/or professional experiences of various pathways to lung cancer diagnoses, to identify delays in diagnosis. We audio-recorded, transcribed, and coded interviews in two stages. First, we conducted deductive coding using three time-period intervals from the Models of Pathways to Treatment framework: appraisal, help-seeking, and diagnostic (i.e., excluding pre-treatment). Second, we conducted inductive coding to identify themes within each time-period interval, and classified these themes as either barriers or facilitators to diagnosis. Coding and thematic summarization were completed independently by two separate analysts who discussed for consensus.

**Results:**

Eight of the patient participants had formerly smoked, and five had never smoked. We identified eight barrier/facilitator themes within the three time-period intervals. Within the appraisal interval, the barrier theme was (1) minimization or misattribution of symptoms, and the facilitator theme was (2) acknowledgment of symptoms. Within the help-seeking interval, the barrier theme was (3) hesitancy to seek care, and the facilitator theme was (4) routine care. Within the diagnosis interval, barrier themes were (5) health system challenges, and (6) social determinants of health; and facilitator themes were (7) severe symptoms and known risk factors, and (8) self-advocacy. Many themes were interrelated, including minimization or misattribution of symptoms and hesitancy to seek care, which may collectively contribute to care and imaging delays.

**Conclusions:**

Interventions to reduce hesitancy to seek care may facilitate timely lung cancer diagnoses. More prompt referral to imaging—especially computed tomography (CT)—among symptomatic patients, along with patient self-advocacy for imaging, may reduce delays in diagnosis.

**Supplementary Information:**

The online version contains supplementary material available at 10.1186/s12875-023-02158-7.

## Background

Lung cancer is the leading cause of cancer mortality in the United States (U.S.), and the second most commonly diagnosed cancer among men and women [1]. 46% of lung cancers are diagnosed at distant stage when five-year survival is 6%, while just 24% of cases are diagnosed at localized stage when five-year survival is 60% [1]. Routine screening can help detect cases at earlier stage, [2] but screening is only recommended for specific groups of non-symptomatic individuals at highest risk for lung cancer [3]. Since 2013, the United States Preventive Services Task Force (USPSTF) has recommended that eligible individuals receive annual screening through low-dose computed tomography (LDCT) [3–5]. Most lung cancer diagnoses occur in individuals who are not screened due to both the lack of eligibility among many based on age and/or smoking history, as well as low screening uptake among those who are eligible [6, 7].

Early-stage lung cancer is typically asymptomatic, presenting challenges to early diagnosis [8]. As lung cancer progresses, symptoms including cough, chest pain, shortness of breath, and weight loss [8, 9] can mimic other diseases (e.g., asthma) or causes (e.g., allergies), or develop in the presence of comorbidities such as chronic obstructive pulmonary disease [8]. In the U.S., patients with persistent or severe acute symptoms are typically referred for chest radiographs (X-rays) as opposed to computed tomography (CT) scans, especially in the absence of other known risk factors [10]. People who never smoked may face delays in obtaining a lung cancer diagnosis. Some studies suggest that limited knowledge of lung cancer symptoms, risk perception, and clinical bias could contribute to diagnosis delays in people who never smoked [11, 12]. Since the incidence rate of lung cancer in people who never smoked is 12 to 30 times lower than the incidence rate among people who currently smoke, [13] it makes sense for providers to have low suspicion of lung cancer upon initial presentation of symptoms in people who never smoked; however, providers should consider lung cancer in the differential diagnosis of symptomatic patients [14].

While some studies have examined patient experiences leading up to a lung cancer diagnosis, [15–20] few studies have examined pathways to lung cancer diagnosis from both patient and provider perspectives [21, 22]. Prior Australia-based studies identified barriers to lung cancer diagnosis including patient rural residence and limited knowledge among some primary care providers regarding how or where to refer patients to specialists, and facilitators including patient social network concern fueling a sense of urgency to seek care [21, 22]. A study in the United Kingdom found that the pathways to lung cancer diagnosis can vary beyond primary care-initiated routes, justifying a need for research to consider differential routes to diagnosis, such as emergency department admissions [23]. Pathways to lung cancer diagnosis may differ among U.S. patients, as primary care-seeking patterns differ from other countries. The specialties of primary care providers vary in the U.S.; for example, 20% of respondents in one survey of American women considered their obstetrician-gynecologist as their primary care provider [24]. Approximately 25% of American adults lack a primary care provider [25]. Past U.S.-based studies conducted among lung cancer patients identified various experiences and care-seeking behaviors prior to a lung cancer diagnosis including symptoms that the patient or their doctor attributed to other causes, and experiencing severe acute symptoms that lead to immediate care seeking, often at an emergency department [19, 26].

**Fig. 1:**
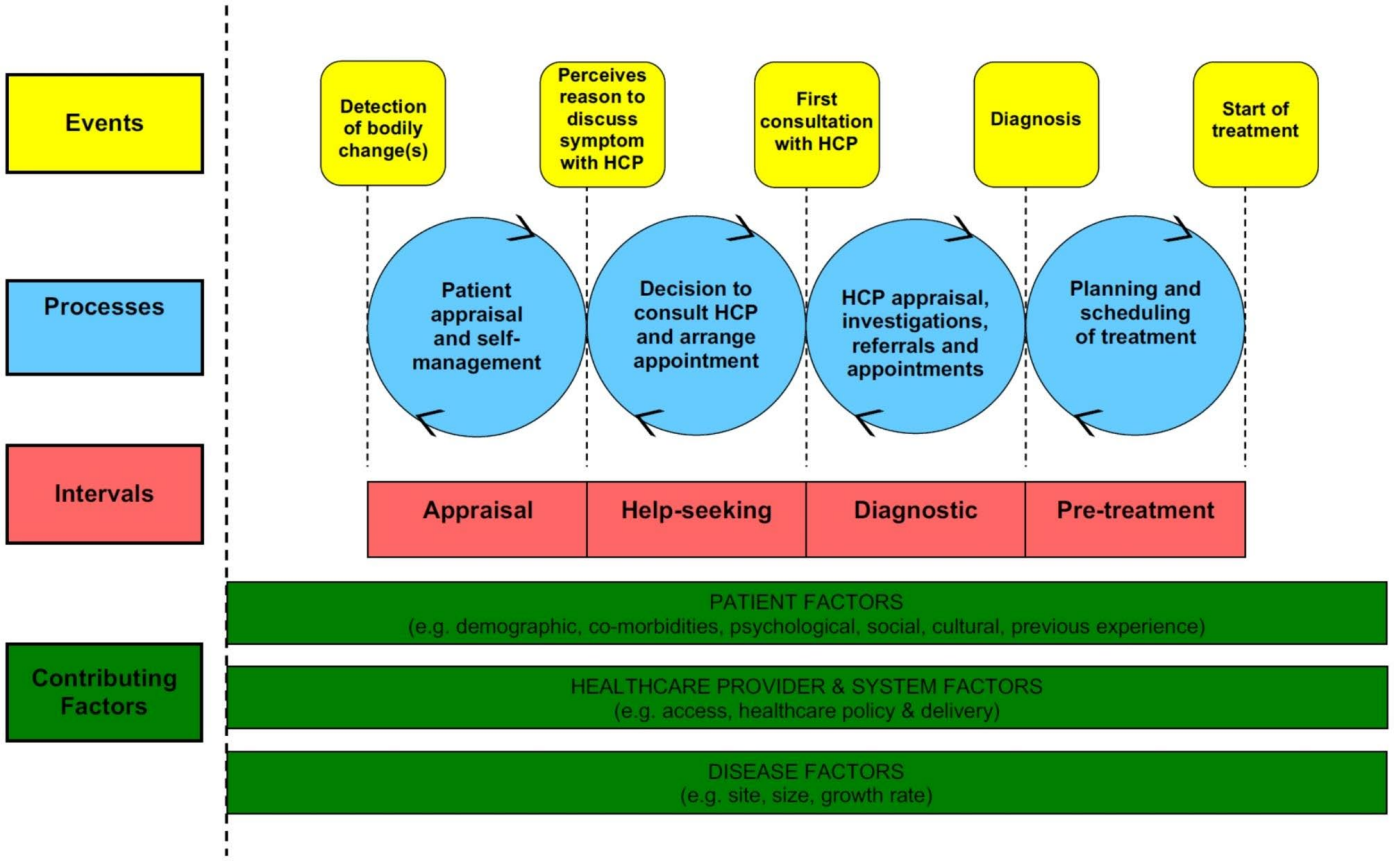
Model of pathways to treatment (MPT) framework used to help define the appraisal, help-seeking, and diagnostic intervals. Reproduced with permission from the publisher and authors of Scott et al., 2012 [28]

Pathways to diagnosis include all of the processes and experiences leading up to a diagnosis. The Model of Pathways to Treatment (MPT) is a framework that characterizes pathways to disease diagnosis and treatment within time intervals. The first three intervals encompass the time periods leading up to and including diagnosis: appraisal, help-seeking, and diagnostic (i.e., excluding pre-treatment) (Fig. 1) [27, 28]. The MPT framework has been used in prior studies of cancer and has helped to identify barriers and facilitators in the pathway to diagnosis [21, 22, 29–32]. In this study, we used the framework to identify themes leading up to a lung cancer diagnosis in a U.S. academic healthcare system. We examined the perspectives of both patients and providers to characterize pathways to lung cancer diagnosis among unscreened individuals to inform opportunities for intervention that could promote earlier detection of lung cancer.

## Methods

### Procedures and participants

This study and all procedures were approved by the Institutional Review Board (IRB) at the University of Utah (IRB #00123466). Lung cancer patients were eligible to participate in the study if they had not received asymptomatic screening, were English-speaking, received care at the University of Utah, and had consented to the Total Cancer Care Study, [33] which enrolls individuals who are diagnosed with any cancer type at the Huntsman Cancer Institute. Study staff identified eligible patients by chart review or physician referral. No patients were excluded based on age, gender, race, ethnicity, stage at diagnosis, or time since diagnosis. Eligible patients were invited to participate through e-mail and a subsequent phone call. We did not restrict based on patient smoking histories when inviting the first batch of participants. After the first batch, we used purposive sampling to preferentially invite those with no or < 30 pack-year smoking histories to target patients ineligible for asymptomatic screening according to the 2013 USPSTF recommendations. Of 35 patients contacted, two declined, 16 did not respond, three set up interviews but did not complete them, and 14 completed interviews. One of the interviewees was later excluded, as their tumor histologic type was identified as likely not lung cancer upon review, leaving a total of 13 completed interviews for analysis.

Providers were eligible to participate in the study if they were a practicing physician in the University of Utah health system. We did not exclude providers based on credentials or specialty in order to assess perspectives from a variety of providers who may be involved in the pathway to lung cancer diagnoses. Study staff identified eligible providers through the University of Utah “Find a Doctor” website or by referral. Eligible providers were invited to participate via e-mail. Of 112 providers contacted, eight declined, 91 did not respond, and 13 completed interviews.

### Data collection

We conducted semi-structured interviews (by authors LG, female Ph.D.; KLM, female M.P.H.; MO, female medical student; and SG, female medical student) between October 18, 2019, and September 7, 2021. Interviews were conducted with patients by telephone and with providers either in-person or by telephone. All interviews began with an informed consent process, and participants were e-mailed a copy of the consent cover letter. Interviews did not have a time limit; interviews were a median of 21 min (range 11–71) with patients and a median of 23 min (range 17–53) with providers. All interviews were audio-recorded and transcribed after the interview using the Microsoft Office 365 transcribe tool, followed by review and corrections by study staff. Participants were not re-contacted post-interview and did not provide feedback on transcripts or findings.

Patient interviews focused on the events leading up to their lung cancer diagnosis, including symptoms, healthcare usage, medications for related symptoms and pre-existing conditions, and diagnostic tests using semi-structured interview questions previously published [34]. Patients were also asked open-ended questions, including, “Could you tell me about your cancer story?” and “What drove you to seek care initially?” Topics also included which types of providers were seen prior to diagnosis. Patient electronic health records were accessed to collect demographic information (sex, age at diagnosis, race, and ethnicity), smoking status, smoking pack-year history among people who ever smoked, cancer histologic subtype, stage at diagnosis, and diagnosis date. Patient characteristics are shown in Supplementary Table 1.

Provider interviews focused on the most common pathway to lung cancer diagnosis from symptoms, if existent, through diagnosis. Providers were asked to respond with respect to their personal or professional experiences, as some providers may have rarely or not knowingly participated in patient referral pathways that resulted in a lung cancer diagnosis. Question topics included presenting symptoms that providers associate with lung cancer, differences in diagnosis pathways between patients with and without a history of smoking, patient behaviors that raise or lower the index of suspicion for lung cancer, and what factors the provider thought may influence a patient’s pathway to diagnosis [34].

### Data analysis

Data were analyzed using a two-phase thematic analysis framework following the applied thematic analysis approach [35]. In the initial deductive phase, the two analysts (authors LG and RDM) coded excerpts from interview transcripts using the appraisal, help-seeking, and diagnostic intervals. Using the MPT framework [27, 28] and the Aarhus statement [36] as frameworks, we defined the intervals using the definitions in Table 1. After each transcript was independently coded, the analysts met to discuss and reach consensus.

**Table 1:** Appraisal, help-seeking, and diagnostic interval defined beginning and end

Interval	Beginning	End
Appraisal	Patient’s detection of bodily changes	Patient perceives a reason to discuss a symptom with a healthcare provider
Help-seeking	Patient’s perception of a reason to consult a healthcare provider	Prior to the first consultation with a healthcare provider
Diagnostic	Patient’s first consultation with a healthcare provider	Lung cancer diagnosis

Once the initial deductive coding phase was completed, the two analysts (authors LG and RDM) independently reviewed the code reports to identify broad themes in each of the first three time-period intervals. The analysts then met to discuss and reach consensus on the themes and to finalize the codebook (Supplementary Table 2). The two reviewers used the codebook to independently code the transcripts, and then met to compare codes and reach consensus. The two reviewers independently examined code reports to classify broad themes as barriers and facilitators to lung cancer diagnosis, then met to discuss and finalize the theme categorization. We summarized the final themes in tables and identified representative quotes. Quotes were lightly edited by removing filler words such as “um,” “like,” and “you know” for concision and clarity. All coding and analyses were conducted in Dedoose Version 9.0.46 [37].

## Results

### Participant demographics

The majority of the 13 patient participants were female (n = 8, 62%) (Supplementary Table 1) and non-Hispanic White (n = 12, 92%). The median age at diagnosis was 66 years (range 44–81). The majority of patients had a history of smoking (n = 8, 62%). While four had smoking histories of < 30 pack years, another four had smoking histories of ≥ 30 pack years. At diagnosis, two resided in rural areas and the others in urban areas. The distribution of stage at diagnosis was: IIB (n = 2, 15%); IIIA (n = 3, 23%); IIIB (n = 1, 8%); and IV (n = 7, 54%). The majority of patients were diagnosed with adenocarcinoma (n = 9, 69%). Other histotypes included large cell neuroendocrine carcinoma, neuroendocrine carcinoma, squamous cell carcinoma, and carcinoma, not otherwise specified (NOS). Eligible patients were diagnosed a median of 2.5 years (range 1.0–7.2) prior to interview.

The 13 provider participants’ specialties were oncology (n = 4), emergency medicine (n = 2), cardiothoracic surgery (n = 2), gastroenterology (n = 2), internal medicine (n = 2), and obstetrics and gynecology (n = 1); the latter two specialties can be considered to provide primary care services [24].

Themes in each of the three initial time-period intervals are summarized in the text with details and supporting quotes included in corresponding tables (Tables 2, 3 and 4).

### Appraisal interval

The appraisal interval encompasses the time period in which patients identify and manage their symptoms. This interval begins when patients detect bodily changes and ends when patients perceive a reason to seek care for their symptoms [27]. In this interval, we identified one barrier, *minimization or misattribution of symptoms* (theme 1), and one facilitator, *acknowledgment of symptoms* (theme 2) (Table 2).

### Theme 1: minimization or misattribution of symptoms

Patients and providers described minimizing or misattributing symptoms to other causes, such as attributing a cough to allergies or asthma, or ignoring unexplained weight loss. Some patients were alarmed by their symptoms, while others were not concerned and viewed their symptoms as minor. For example, one patient described decreased energy levels and concentration that persisted for months, but this did not lead them to want to seek care. Providers noted that the non-specific nature of many symptoms associated with lung cancer can cause patients to self-manage their symptoms, as patients are likely to misattribute symptoms to less serious causes.

### Theme 2: acknowledgment of symptoms

Some patients described initially ignoring symptoms until they persisted and became severe enough to affect their quality of life, including their ability to work, before actively acknowledging symptoms as a reason to seek care. Patients and providers described the role that one’s social network can play in appraising and acknowledging symptoms, such as others noticing a cough or a decrease in energy. Providers described how patient or social network appraisal of symptoms as a problem can lead to a desire to seek care.

**Table 2:** Themes and participant quotes in the appraisal interval

Themes	Patients	Providers
Barriers		
Minimization or misattribution of symptoms	“I’ve always had really, really bad allergies, so I blamed a lot on the allergies.” (Patient #12, 44-year-old female, Stage IV, Never smoking history)	“Why would you think you have lung cancer, if you have back pain, or if you just [have] allergy symptoms?” (Provider #12, Lung oncology)
	“I was not doing anything differently. I was not eating better. I was not exercising more significantly… just thought that the stars were aligned and I (was losing weight)” (Patient #12, 44-year-old female, Stage IV, Never smoking history)	“If you’re not educated to look for symptoms, you don’t understand that your cough or your weight loss isn’t necessarily just lack of food.” (Provider #4, Gastroenterology)
	“My energy level was not there, and my concentration was not there, I was having a heck of a hard time concentrating… that was probably going on for about 4 months before.” (Patient #4, 65-year-old male, Stage IIIB, 40 pack-year current smoking history)	
	“I just wish I could have been told earlier…if I [had] a persistent cough, go get checked because that is a sign of cancer.” (Patient #1, 61-year-old female, Stage IVB, Never smoking history)	
Facilitators		
Acknowledgment of symptoms	“I was coughing so much and losing a lot of weight. I lost 30 pounds” (Patient #13, 66-year-old male, Stage IV, Never smoking history)	“Then sometimes when you probe the patients a little bit further, especially the ones that don’t want to come in, they’ll say, ‘I knew something was wrong,’ or ‘I didn’t want to know’, or ‘my family member actually made [me] come in.’” (Provider #2, Gynecologic oncology)
	“That’s when I decided to go in. It was affecting my job and I couldn’t do the things that I was doing.” (Patient #8, 54-year-old male, Stage IV, 20 pack-year former smoking history, quit 14 years prior to diagnosis)	
	“It was just a nagging cough…and my daughter-in-law did say something like I just don’t like the sound of that.” (Patient #7, 67-year-old female, Stage IIIA, 20 pack-year former smoking history, quit 27.5 years prior to diagnosis)	
	“I felt normal, but…I owned a business and a couple of my employees [had] noticed that my attitude, my go-get- ‘em attitude seemed to be getting less.” (Patient #6, 60-year-old male, Stage IV, Never smoking history)	

### Help-seeking interval

The help-seeking interval is the time period when patients perceive a reason to seek care and ends at the first visit with a healthcare provider [27]. This interval can include the time period when a patient chooses to avoid or delay care-seeking after appraising a symptom that they feel may warrant care. In this interval we identified one barrier, *hesitancy to seek care* (theme 3), and one facilitator, *routine care* (theme 4) (Table 3).

### Theme 3: hesitancy to seek care

Patients and providers both described that patients may be hesitant to seek care for various reasons, including denial or fear of being diagnosed with a serious health problem. One patient described being reluctant to contact their doctor as they were afraid that they were overreacting to their symptoms. Because many of the interviews took place during 2020 and 2021, some patients and providers also described delayed care due to fear of COVID-19 exposure.

### Theme 4: routine care

Patients and providers described diagnostic workups occurring after patients mentioned a symptom during a routine care visit or seeking care for a symptom from their primary care provider. One provider described how patients who are regularly seeing a doctor are more likely to report symptoms, be referred for imaging, and receive a diagnostic work-up. Another provider explained how patients who primarily seek care from emergency departments and urgent care facilities may be less likely to have a chronic condition or malignancy diagnosed, as they do not have ongoing care from a primary care provider who has a longitudinal view of their health condition(s).

**Table 3:** Themes and participant quotes in the help-seeking interval

Themes	Patients	Providers
Barrier		
Hesitancy to seek care	“I always had this fear like I was overreacting to things, like ‘I don’t really need to be seen for that. I’m being crazy.’” (Patient #12, 44-year-old female, Stage IV, Never smoking history)	“…You’ve really got two different populations. [There is] the population of patients that pops out and wants to get seen and scanned right away, and then you have the other side of the coin where they’re like ’I don’t want to know,’ [or] ‘I am afraid of what they’re going to tell me,’ so they don’t report their symptoms, or they don’t come in…” (Provider #2, Gynecologic oncology)^a^
		“We actually are seeing this year that our numbers are down, for peripheral lung cancers, and we think it’s because…people are not going to the [emergency department] (ED) because there are problems here with [COVID-19].” (Provider #7, Cardiothoracic surgery)
Facilitator		
Routine care	“I developed a typical cough, which I thought was probably just a normal cough. And I figured it would go away in a couple of weeks and it didn’t. So, in about August, I decided to go to the doctor, my general practitioner.” (Patient #6, 60-year-old male, Stage IV, Never smoking history)	“Patients who are medically literate, and are seeing a doctor on a regular basis, are much more likely to have a chest X-ray, or to report symptoms that might be concerning and prompt a workup.” (Provider #10, Emergency medicine)
		“Individuals who are high utilizers of emergency rooms and urgent care but don’t have consistent primary or internal medicine care…[have] another risk factor [because] if you’re seeing a variety of different providers for just acute concerns, it doesn’t necessarily raise the flag of which to delve further into a chronic condition or a malignancy.” (Provider #9, Obstetrics and gynecology)
		“There are some patients that [the emergency department] is their only option for healthcare. So, we see [them] more often because they come in for everything. The other patients who get excellent primary care and specialty care outpatient, we only seem to [see them when they] have more of a crisis.” (Provider #6, Emergency medicine)

^a^This quote was previously published in Lawson-Michod et al. [34]

### Diagnostic interval

The diagnostic interval is the time period in which a healthcare provider assesses the patient, investigates, and makes referrals to specialists. The interval begins with the first consultation with a healthcare provider and ends when the patient obtains a diagnosis [27]. In this interval, we identified two barriers, *health system challenges* (theme 5) and *social determinants of health* (theme 6), and two facilitators, *severe symptoms and known risk factors* (theme 7) and *self-advocacy* (theme 8) (Table 4). In addition to the diagnostic pathways captured by these themes, some patients had their lung cancers identified incidentally from imaging performed for reasons unrelated to lung cancer symptoms.

### Theme 5: health system challenges

Patients and providers described challenges that resulted in missed opportunities for earlier diagnosis at the facility level. These challenges include managing the time it takes to schedule multiple doctor appointments when juggling a busy schedule. Systemic issues may have played a role in some diagnostic delays, as one provider described how Medicare will not cover LDCT for people who never smoked. Another provider noted that patients who present to emergency departments are more likely to get CT scans than patients seeking care from primary care providers. In addition, providers described challenges in triaging care and treating the whole body within the limited time that providers have to see patients.

### Theme 6: social determinants of health

Patients and providers described how social determinants of health, including decreased access to healthcare and low socioeconomic status, can lead to a longer time to diagnosis. Patients and providers also described that living in more rural areas may present barriers to obtaining a timely diagnosis, such as limited access to medical specialists and imaging equipment causing scheduling delays and/or necessitating patient travel to access care. Providers described how patient distrust in the healthcare system that may vary by race and ethnicity can create a barrier to diagnosis. Social support, including the ability to take off work and obtain childcare that allows patients to attend healthcare appointments, can also impact the ability to receive a diagnosis in a timely manner.

### Theme 7: severe symptoms and known risk factors

Patients and providers described how severe symptoms, such as hemoptysis, weight loss, shortness of breath, and chronic cough, or known risk factors, particularly smoking history, secondhand smoke exposure, or occupational exposures, can increase providers’ suspicion of lung cancer and lead to referral for imaging. Providers also described how a history of smoking influences the diagnostic work-up, as the suspicion of lung cancer is much higher in people who ever smoked than in people who never smoked.

### Theme 8: self-advocacy

Patients and providers described how trusting one’s intuition regarding bodily changes, self-advocacy and persistence can lead to referrals for imaging and facilitate a diagnosis. Providers conveyed that some patients must advocate for themselves with respect to their symptoms to be referred to imaging, including one provider who described how patients sometimes must be persistent with seeking care to obtain a diagnosis.

**Table 4:** Themes and participant quotes in the in the diagnostic interval

Themes	Patients	Providers
Barriers		
Health system challenges	“I was told before a pulmonologist would see me, I needed a chest X-ray, and I needed a pulmonary function test…I remember being really irritated and I blew it off because I was like I don’t have time for that…all I was imagining was all these appointments I had to schedule.” (Patient #12, 44-year-old female, Stage IV, Never smoking history)	“I don’t think [never smokers] get scanned as much or images much, rather CT or chest X-ray… also [for] the current low dose CT screening scans, Medicare will only pay for them if you have a history of smoking.” (Provider #7, Cardiothoracic surgery)
	“They saw these different signs in my lungs, and I was terrified… and I kept contacting them and they were very patient with me. They were able to get [me in] for a lung biopsy.” (Patient #2, 71-year-old female, Stage IVA, 10–20 pack-year former smoking history, quit 25 years prior to diagnosis)	“Primary care doctors are trained to say ‘hey let’s triage this…’ and not just scan everybody all the time. I feel like if a patient gets to the [emergency room] (ER), they’re almost guaranteed to [get a] scan.” (Provider #2, Gynecologic oncology)^a^
	“I had some difficulty when I laid on my right side…I couldn’t breathe as well, so I asked my doctor about it. He sent me to a lung specialist…And the lung specialist did some breathing tests… where you breathe in in a little chamber and…they test your oxygen intake …He also did a lung X-ray and he found nothing. Uhm, which of course we know that lung cancer can’t be found on an X-ray anyway.” (Patient #3, 66-year-old female, Stage IIIB, Never smoking history)	“[A primary care provider] is worried about the whole body…and there are also time constraints…They have what, 15 min per visit? That slight shortness of breath that you get while walking up the hill probably is going to be lower on the totem pole than the knee pain that you’re having.” (Provider #7, Cardiothoracic surgery)
Social determinants of health	“It gets a little dicey in the first month or two of each year when I have to pay the (insurance) out of pocket down” (Patient #6, 60-year-old male, Stage IV, Never smoking history)	“We find that [for] patients…that are maybe hours from a major university hospital just getting their care and getting the appropriate testing takes longer. ” (Provider #8, Cardiothoracic surgery)
		“…Socioeconomic status [and] education [are] all variables that indicate an individual’s ability to obtain a diagnosis in a timely fashion. Along with that, would be social support, [the] ability to take off work, or obtain childcare to go to your healthcare appointment, I think it’s trust in the system. In this case, distrust, especially with regards to racial and ethnic variations in trust in the healthcare system.” (Provider #13, Gastroenterology)
Facilitators		
Severe symptoms and known risk factors	“She said, ‘<<patient name > > I believe it’s just the lisinopril (side effect of medication). But because you smoked for 40 years, we’re going to send you for a chest X-ray, just in case.’ And so, I went to get the chest X-ray… <<provider name > > called me the next day. She told me that I had a tumor.” (Patient #9, 66-year-old female, Stage IIIA, 40 pack-year former smoking history, quit 5.5 years prior to diagnosis)	“Hemoptysis is the is the firm answer that says it’s probably lung cancer and it’s probably really bad.” (Provider #11, Lung oncology)
		“I have had a few patients I was worried [about]…and fortunately they ended up not having lung cancer, but the first thing for me would be risk factors, if they have a history of smoking and then that combined with cough and weight loss then I would say I’m worried about you and we need to work that up.” (Provider #5, Neurology)
		“Nonsmokers get diagnosed in a much more advanced stage than smokers because our index of suspicion is higher [for smoked]…” (Provider #12, Lung oncology)
Self-advocacy	“I had my annual physical…and my doctor said that I had blood in my urine. And I said, you told me this the last couple years. Could we check this a little further? I said I want to know what’s going on. He goes well, sometimes there’s a little blood in urine like that. It’s not a big deal…so this time I said let’s check a little further and look into it.” (Patient #3, 66-year-old female, Stage IIB, Never smoking history)	“They’re like, ‘I had to advocate for myself because I kept having symptoms and nobody would scan me and so we see that a lot of people are like I was short of breath…I had chest discomfort. I was losing weight and then finally somebody decided to scan me.’” (Provider #2, Gynecologic oncology)
	“I was having some discomfort in my right sternum and back under my shoulder blade. And I kept mentioning it…[a provider] called me the next day and told me that she pulled previous films…and she said that that’s where she noticed the lesion.” (Patient #5, 81-year-old male, Stage IIB, 30 pack-year former smoking history, quit 14 years prior to diagnosis)	“I think if a patient continues to seek help and not give up because they didn’t get help, that may lead to, hopefully, a sooner diagnosis” (Provider #4, Gastroenterology)
	“I went to the doctor for my physical and I said, the only thing I worry about at this point is cancer…I was pushing her…I knew the right things to say to get her to say OK. ‘OK,’ she said. ‘We’ll do a test. We’ll do an MRI…CAT scan,’ I don’t know what it was. And then, as she’s looking at my chart, she says ‘ …as long as we’re doing that, let’s do the chest X-ray.” (Patient #10, 77-year-old female, 6 pack-year former smoking history, unknown years since quitting)	

^a^This quote was previously published in Lawson-Michod et al. [34]

## Discussion

This study examined barriers and facilitators to lung cancer diagnosis across the appraisal, help-seeking, and diagnostic intervals. Our study builds upon previous studies [15, 16, 19–21, 23] as, to our knowledge, it is the first qualitative study in the U.S. that simultaneously considered patient and provider perspectives. We report that emerging themes were similar to those identified in prior qualitative studies conducted among patients and providers outside of the U.S [15–18, 21]. and in patient-only studies conducted in the U.S [19, 26]. A key finding in this study was that themes identified between patients and providers largely aligned. Prior studies, and ours, identified barriers that may delay a diagnosis, including minimization or misattribution of symptoms, hesitancy to seek care, health system challenges, and social determinants of health. Some themes that were focused on the patient-level, such as acknowledgment of symptoms and minimization or misattribution of symptoms, may reflect system-level effects such as health literacy. The patient narratives, combined with perspectives from providers, can help to identify opportunities for interventions at the patient, provider, and health-system levels to facilitate timely diagnosis and improve patient outcomes.

The majority of lung cancers are diagnosed in unscreened individuals among both individuals who are eligible and individuals who are ineligible for screening [7]. A number of factors can contribute to delays in obtaining a lung cancer diagnosis, including typical lung cancer presentation and barriers at the individual- and system-level. While providers noted that many patients have no symptoms, they also noted that commonly described severe or alarming symptoms were hemoptysis, weight loss, shortness of breath, and chronic cough. A prior systematic review of symptomatic lung cancer diagnoses found insufficient evidence of symptoms as strong predictors of lung cancer diagnosis, apart from hemoptysis [38]. This is consistent with our study, as some patients and providers described the absence of symptoms or presentation of non-specific symptoms that can mimic or co-occur with other respiratory conditions.

It is well established that individuals who never smoked face more delays in symptom appraisal and diagnosis compared with individuals with a smoking history because they are known to have a lower risk of lung cancer [11]. We observed this in our study, as patients without a history of smoking did not consider lung cancer as a possible reason for their symptoms early on and providers described how individuals without a history of smoking are less likely to be referred for imaging, which is typically the last step in obtaining a lung cancer diagnosis. In addition, previous qualitative studies conducted in Australia [21] and New Zealand [16] observed delays at the health system-level, including delayed referral to specialists. Health system challenges, including delays and difficulty obtaining CT imaging, also emerged as a barrier in our study. Our study was also consistent with prior research reporting that lung cancer diagnosis occurs through multiple routes including routine care as well as presenting in the emergency department [23]. Previous studies have also reported the importance of social networks in appraising symptoms and advocating for care, [15, 21] consistent with patient and provider-reported themes in our study.

### Strengths and limitations

Strengths of this study include our ability to examine the experiences leading up to a lung cancer diagnosis in a patient’s own words, and the fact that the patient sample was diverse in age at diagnosis, sex, cancer stage, and histotype. The majority of interviews were conducted during the height of the COVID-19 pandemic and had to be conducted over the telephone. This may have contributed to interviews being brief and may have limited the depth of responses. While we observed a repetition of themes across our data, we did not formally assess for data saturation [39]. It is possible that additional interviews could have elicited new themes related to our research question. Providers were diverse in their medical specialties, which provided a range of perspectives offering important insights as healthcare seeking patterns vary widely in the U.S. However, selection bias may be a limitation as providers with a specific interest in lung cancer may have been more motivated to participate. A limitation of this study was the lack of racial and ethnic diversity among participants, as 12 out of 13 patients identified as non-Hispanic White. As with all qualitative research, social desirability and recall biases likely influenced how participants described their experiences and perspectives. Findings from this research may not be generalizable to patients in other healthcare settings, though we believe that the themes likely resonate across populations and settings. In addition, future research is needed to determine whether pathways to lung cancer diagnosis in unscreened individuals differ between those who are eligible for asymptomatic screening and those who are ineligible.

## Conclusion

The large proportion of lung cancers that are diagnosed among non-screened patients [6, 7] presents a need for better understanding of the pathways to diagnosis in unscreened individuals. This research identified key barriers and facilitators to lung cancer diagnosis throughout the appraisal, help-seeking, and diagnostic intervals. Minimization or misattribution of symptoms, hesitancy to seek care, access and system issues, distrust in healthcare, and rural residence continue to pose barriers to obtaining a diagnosis for some individuals. Interventions to promote patient appraisal and facilitate earlier help-seeking among individuals with symptoms may lead to faster diagnosis, particularly among individuals without a history of smoking. Potential interventions for shortening diagnosis pathways include improving patient knowledge of lung cancer symptoms and awareness of risks based on smoking history and other exposures. Patient self-advocacy for investigation of symptoms may lead to more timely imaging referral. Because X-rays have low sensitivity for lung cancer diagnosis in symptomatic individuals, it has been suggested that after negative X-ray imaging and continued symptoms, referral for CT scans should be considered by healthcare providers [40]. Further research is needed to examine how pathways to lung cancer diagnosis vary among other populations including among different racial and ethnic groups, across different healthcare and insurance systems, and across socioeconomic strata.

### Electronic supplementary material

Below is the link to the electronic supplementary material.

Supplementary Material 1

## Data Availability

The interview transcripts for this study contain potentially identifiable information and are not sharable as they would compromise participant privacy and anonymity. All quotes used in analyses with extracted protected health information and identifiable information are available from the corresponding author upon reasonable request.

## Abbreviations

MPT: Models of Pathways to Treatment
U.S.: United States
LDCT: Low-dose computed tomography
CT: Computed tomography
USPSTF: United States Preventive Services Task Force
