# Supplementary material for: Pathways to lung cancer diagnosis among individuals who did not receive lung cancer screening: a qualitative study
Source: BMC Prim Care. 2023 Oct 3;24:203. doi: 10.1186/s12875-023-02158-7 (PMC10548694; doi:10.1186/s12875-023-02158-7)
Supplement: Supplementary file 1 — Supplementary Material 1 [file 12875_2023_2158_MOESM1_ESM.docx]

| **Supplementary Table 1. Lung Cancer Patient Characteristics** | | | | | | | | | |
| --- | --- | --- | --- | --- | --- | --- | --- | --- | --- |
| **Patient #** | **Age at diagnosis, race and ethnicity, and sex** | **Smoking** | **Stage and histology** | **RUCA code class** | **Routine health-care user** | **Referral pathway** | **First symptom/s*** | **Providers consulted** | **Time from diagnosis to interview (yr)** |
| 1 | 61yo NHW F | NS | IVB, Adeno | Urban | Yes | Bone biopsy and other imaging (PET) after post-fall repeat knee replacement | Persistent cough | -- | 1.0 |
| 2 | 71yo NHW F | FS: 10-20 PY, 25 yr since quit | IVA, NEC NOS | Urban | No | Intentional imaging for nodules found at ED visit (CT, watch and wait until biopsy) | **Severe chest pain** | ED (CT), pulmonologist (x3, CT), ED (unclear with or without imaging), pulmonologist (biopsy) | 2.5 |
| 3 | 66yo NHW F | NS | IIB, Mucinous adeno | Urban | Yes | Other imaging for blood in urine (CT, urology) | **Pain on right side, shortness of breath** | PCP, pulmonologist (X-ray), PCP, urologist (CT), oncologist (before 2nd opinion) | 2.7 |
| 4 | 65yo NHW M | FS: 40 PY, quit at diagnosis | IIIB, Squamous cell carcinoma | Urban | Yes | Intentional imaging (X-ray) | Persistent cough, fatigue, **pneumonia** | PA, imaging (X-ray) | 1.7 |
| 5 | 81yo NHW M | FS: 30 PY, 14 yr since quit | IIB, Adeno | Urban | Yes | Other imaging for COPD (CT, cardiology during workup for WATCHMAN device) | Pain in right sternum and under shoulder | -- | 2.4 |
| 6 | 60yo NHW M | NS | IV, Adeno | Urban | No | Intentional imaging (X-ray) | Low mood observed by social network, **persistent cough** | PCP, imaging (X-ray), pulmonologist | 6.4 |
| 7 | 67yo NHW F | FS: 20 PY, 27.5 yr since quit | IIIA, Adeno | Rural | Yes | Other imaging (CT, urology) | **Persistent cough** | PCP (x3, X-ray), thyroid specialist, allergist (bronchoscopy), PCP (X-ray), urologist (CT) | 2.5 |
| 8 | 54yo HW M | FS: 20 PY, 14 yr since quit | IV, Carcinoma NOS | Urban | No | Other imaging (X-ray) | **Bone mass in right shoulder (pain)**, hip pain, weakness, shortness of breath | Urgent care, physical therapist (x3), neurologist (X-ray) | 7.2 |
| 9 | 66yo NHW F | FS: 40 PY, 5.5 yr since quit | IIIA, Mucinous adeno | Rural | Yes | Intentional imaging (X-ray) | Fatigue, **light cough** | PCP, imaging (X-ray) | 2.1 |
| 10 | 77yo NHW F | FS: 6 PY, unknown yr since quit | IVA, Adeno | Urban | Yes | Intentional imaging for known spots from bronchoscopy (X-ray) | **Heavy cough that cleared by time of bronchoscopy**, fatigue | PCP, pulmonologist (bronchoscopy), PCP, imaging (X-ray) | 1.6 |
| 11 | 68yo NHW F | FS: 78 PY, quit at diagnosis | IIIA, Large cell NEC | Urban | Yes | Intentional imaging for regular pneumonia (CT) | Bronchitis | Pulmonologist, imaging (CT) | 4.8 |
| 12 | 44yo NHW F | NS | IV, Adeno | Urban | No | Intentional imaging for allergy and asthma symptoms (X-ray) | Heavy recurring cough, weight loss, **bronchitis** | PCP (x3), imaging (X-ray), hospital (lung function), pulmonologist, imaging (CT) | 5.2 |
| 13 | 66yo NHW M | NS | IV, Adeno | Urban | Yes | Intentional imaging (X-ray; later CT) | **Worsening heavy cough**, difficulty breathing, fatigue, weight loss | PA, imaging (X-ray), PCP, imaging (CT) | 5.2 |
| *First symptom/s = **bold** indicates the reason for seeking help, if any  Abbreviations: Non-Hispanic White (NHW), Hispanic White (HW), Female (F), Male (M), Years old (yo); Formerly smoked (FS), Never smoked (NS), Pack years (PY), Years (yr); Adenocarcinoma (Adeno), Neuroendocrine carcinoma (NEC), Not otherwise specified (NOS); Rural-Urban Commuting Area (RUCA); Chronic obstructive pulmonary disease (COPD), Computed topography scan (CT)  Positron emission tomography scan (PET); Emergency department (ED), Physician assistant (PA), Primary care physician (PCP); Months (mo) | | | | | | | | | |

| Supplementary Table 2. Themes and codes within MPT intervals | | |
| --- | --- | --- |
| MPT Interval | **Themes** | **Codes** |
| Appraisal | 1. Minimization or misattribution of symptoms | - - Not alarmed   - Health literacy |
|  | 2. Acknowledgment of symptoms | - - Alarmed     - Family friend   - Proactive patients |
| Help-seeking | 3. Hesitancy to seek care | - - Hesitant patients   - Not alarmed (patients and providers) |
|  | 4. Routine care | - - Routine care/casual   - Intentionally seeking care |
| Diagnostic | 5. Health system challenges | - - Healthcare delay   - Misattributed symptom |
|  | 6. Social determinants of health | - - Distrust in healthcare   - Socioeconomic status   - Rural |
|  | 7. Severe symptoms and known risk factors | - - Severe/alarming symptoms   - Patient lifestyle factors/exposures |
|  | 8. Self-advocacy | - - Self-advocacy   - Referral for imaging |
